# The Relationship between *XRCC1* and *XRCC3* Gene Polymorphisms and Lung Cancer Risk in Northeastern Chinese

**DOI:** 10.1371/journal.pone.0056213

**Published:** 2013-02-08

**Authors:** Shujie Guo, Xiaobo Li, Min Gao, Yuqiong Li, Bei Song, Wenquan Niu

**Affiliations:** 1 State Key Laboratory of Medical Genomics, Rui Jin Hospital, School of Medicine, Shanghai Jiao Tong University, Shanghai, China; 2 Department of Respiratory Medicine, The Fourth Affiliated Hospital of Harbin Medical University, Harbin, Heilongjiang, China; United States of America

## Abstract

**Background:**

The prevalence of lung cancer in China will be the world's highest if allowed to proceed uncurbed. To unravel its genetic underpinnings, we sought to investigate the association of three well-characterized nonsynonymous polymorphisms in *XRCC1* (Arg194Trp and Arg399Gln) and *XRCC3* (Thr241Met) genes with lung cancer risk in northeastern Chinese.

**Methodology/Principal Findings:**

This study was hospital-based in design, encompassing 684 patients with lung cancer and 604 cancer-free controls. Genotyping was performed using the PCR-LDR (ligase detection reactions) method. Data were analyzed by R language and multifactor dimensionality reduction (MDR) software. Single-locus analysis identified significance in genotype distributions of polymorphism Arg194Trp (P = 0.002) and Arg399Gln (P = 0.017), and in allele distributions of Thr241Met (P = 0.005). Carriers of 399Gln/Gln genotype conferred a 147% increased risk relative to the non-carriers (odds ratio (OR): 2.47; 95% confidence interval (95% CI): 1.48–4.13; P<0.001). For Thr241Met, significance persisted under allelic (OR = 1.63; 95% CI: 1.14–2.33; P = 0.005), additive (OR = 1.64; 95% CI: 1.16–2.32; P = 0.005) and dominant (OR = 1.67; 95% CI: 1.17–2.38; P = 0.004) models. However, common allele combinations were comparable in frequency between patients and controls. In interaction analysis, the overall best MDR model included Arg399Gln and Thr241Met polymorphisms, with a maximal testing accuracy of 63.18% and a maximal cross-validation consistency of 10 out of 10 (P = 0.0175).

**Conclusions:**

Our study significantly demonstrated an independent and synergistic contribution of *XRCC1* Arg399Gln and *XRCC3* Thr241Met polymorphisms to lung cancer susceptibility in northeastern Chinese.

## Introduction

It is estimated that China will have the world's highest prevalence of lung cancer, with its mortality rate projected to exceed one million by 2025 if allowed to proceed uncurbed [1]. Smoking and exposure to ionizing radiation constitute the common incentives of lung cancer, and they are also regarded as triggering factors for DNA damage. Converging lines of evidence suggest that cancer can be initiated by DNA damage, which if not repaired, can cause errors during DNA synthesis. Therefore individuals with an inherited impairment in DNA repair capability are often at elevated risk of developing cancer [2]. Most DNA damage can be removed by DNA repair enzymes, and thereof X-ray repair cross-complementing protein 1 and 3 (XRCC1 and XRCC3) are listed as two promising candidates.

Both biological and biochemical data indicate a direct role of XRCC1 and XRCC3 in DNA repair. Specifically, XRCC1 can stimulate the DNA kinase at damaged DNA termini, and thereby accelerate the overall repair reaction [3]. In human fibroblasts, XRCC1, interacting with DNA ligase III, was found to localize with nucleotide excision repair components [4]. Contrastingly, XRCC3 function was not limited to initiate homologous recombination, but extended to later stages in formation and resolution of the intermediates, possibly by stabilizing heteroduplex DNA [2]. Despite the strong biological rationale for the involvement of XRCC1 and XRCC3 in DNA repair or stabilization, recent findings from genome-wide association studies on lung cancer failed to detect any positive signals in or flanking their coding genes. Although the candidate gene approach, which deals with prespecified genes that are thought to partake disease pathophysiology, may not replace the genome-wide approach, it is an important alternative strategy to unravel the genetic underpinnings of complex disease [5].

In this study, we sought to investigate the association of three well-characterized nonsynonymous polymorphisms in *XRCC1* (rs1799782∶Arg194Trp and rs25487∶Arg399Gln) and *XRCC3* (rs861539∶Thr241Met) genes with risk of lung cancer in a northeastern Chinese population.

## Methods

### Study population

This study was hospital-based in design and included a total of 1286 participants of Chinese descent as previously reported [6], [7]. In detail, all subjects were recruited from three hospitals in Harbin city, Heilongjiang province, and they were local residents of Han descent. All participants were underwent either the computed tomography (CT) or enhanced CT or positron emission computed tomography (PET)-CT scan, which was confirmed by clinical doctors of respiratory medicine. Those who were susceptible to lung cancer were further pathologically confirmed by biopsy, and those with normal CT or enhanced CT or PET-CT results were treated as cancer-free controls in this study. Lung cancer was clinically classified into squamous cell cancer, adenocarcinoma, and small cell cancer.

The lung cancer group involved 684 sporadic patients aged 57.24 (standard deviation: 9.84) years. The remaining participants (n = 602) formed age-matched (56.8 (9.95) years) cancer-free controls. This study had protocols approved by the Ethics Committee of Harbin Medical University, and was conducted according to the Declaration of Helsinki Principles. All participants signed the informed written consent.

### Demographic characteristics

At enrollment, age and gender were recorded according to a self-designed questionnaire. Meanwhile, the status of cigarette smoking and alcohol drinking was also defined. Smoking was categorized as never, ever or current smoking (at least one cigarette per day). Drinking was categorized as never, ever or current drinking. Here, current drinking referred to consumption of at least one alcoholic drink during the past 30 days.

### Genotype determination

2 mL venous blood was taken from each participant and genomic DNA was extracted from white blood cells using TIANamp Blood DNA Kit (Tiangen Biotect (Beijing) Co., China). Genotypes of the examined polymorphisms were determined by using the PCR-LDR (polymerase chain reaction-ligase detection reactions) method by ABI 9600 system (Applied Biosystems, USA) [8]. Amplification parameters were 94°C for 2 min, 35 cycles of 94°C for 15 s, 60°C for 15 s, 72°C for 30 s, and a final extension step at 72°C for 5 min. Two specific probes and one common probe were synthesized for each polymorphism. The common probe was labeled at the 3' end with 6-carboxy-fluorescein and phosphorylated at the 5' end. The reacting conditions of LDR followed 94°C for 2 min, 20 cycles of 94°C for 30 s and 60°C for 3 min. After reaction, 1 µL LDR reaction products were mixed with 1 µL ROX passive reference and 1 µL loading buffer, and then denatured at 95°C for 3 min, and chilled rapidly in ice water. The fluorescent products of LDR were differentiated using ABI sequencer 377 (Applied Biosystems, USA).

### Statistical analysis

Between-group comparisons were made using unpaired t-test for continuous variables and χ^2^ test for categorical variables. Hardy-Weinberg equilibrium was checked on a contingency table of observed-versus-predicted genotype distributions by χ^2^ test or Fisher's exact test. Logistic regression analysis was adopted under assumptions of allelic, additive, dominant and recessive models of inheritance, respectively. Statistical significance was declared at P<0.05.

Frequencies of allele combinations were estimated by haplo.em program, and odds ratio (ORs) and 95% confidence interval (CI) were estimated by haplo.cc and haplo.glm programs according to a generalized linear model [9]. The haplo.em, haplo.cc and haplo.glm were implemented using Haplo.stats software (version 1.4.0) developed by the R language (http://www.r-project.org/). Study power was calculated using PS (Power and Sample Size Calculations) software (version 3.0).

Analysis on the interaction of examined polymorphisms was carried out in the open-source multifactor dimensionality reduction (MDR) software (version 2.0) (www.epistasis.org) [10], [11]. All possible combinations of one to three polymorphisms were constructed using MDR constructive induction. A Bayes classifier in the context of 10-fold cross-validation was used to estimate the testing accuracy of each best model. A single best model had maximal testing accuracy and cross-validation consistency, which measures the number of times of 10 divisions of the data that the best model was found. Statistical significance was evaluated using a 1000-fold permutation test to compare observed testing accuracies with those expected under the null hypothesis of null association. Permutation testing corrects for multiple testing by repeating the entire analysis on 1000 datasets that are consistent with the null hypothesis.

## Results

### Baseline characteristics

Patients and controls shared similar age distributions (P = 0.776). Male gender was significantly higher in patients than in controls (P = 0.013), so was the prevalence of current smoking (P<0.005) or drinking (P<0.005). Among lung cancer patients, those with adenocarcinoma, squamous cell cancer, small cell cancer, and unspecified lung cancer accounted for 37.54%, 32.26%, 20.83%, and 9.38%, respectively. The baseline characteristics of study population were described in Table 1.

**Table 1:** The baseline characteristics of study population.

Characteristics	Patients (n = 684)	Controls (n = 602)	P^**^
Age (years)	57.24 (9.84)	56.80 (9.95)	0.776
Sex (male)	72.78%	66.49%	0.013
Smoking			
Current	28.22%	6.99%	
Ever	8.04%	0.54%	<0.005
None	63.74%	92.47%	
Drinking			
Current	15.23%	5.38%	
Ever	1.61%	2.69%	<0.005
None	83.16%	91.94%	
Lung cancer type			
Squamous cell cancer	32.26%	−^*^	
Adenocarcinoma	37.54%	−	
Small cell cancer	20.83%	−	
Unspecified	9.38%	−	

Data are expressed as mean (standard deviation or SD) or percentage as indicated. * data not available. **P values were calculated by using unpaired t-test for age, and by χ^2^ test for other categorical characteristics.

### Single-locus analysis

The genotype distributions of three examined polymorphisms complied with Hardy-Weinberg equilibrium in both patients and controls (P>0.05). As shown in Table 2, there were significant differences in the genotype distributions of Arg194Trp (P = 0.002) and Arg399Gln (P = 0.017), and in the allele distributions of Thr241Met (P = 0.005). Based on power calculation, the present study of 684 patients and 602 controls had 80.2% power to detect a significant allelic association for Arg399Gln.

**Table 2:** Genotype distributions and allele frequencies of examined polymorphism between patients and controls, as well as their prediction for lung cancer risk.

Gene & polymorphism		Patients (n = 684)	Controls (n = 602)	P_χ2_	Model	OR; 95% CI; P
*XRCC1* gene	Arg/Arg	314	265		Additive	0.95; 0.8–1.12; 0.511
Arg194Trp	Arg/Trp	302	274	0.79	Dominant	0.93; 0.74–1.15; 0.497
(rs1799782)	Trp/Trp	68	63		Recessive	0.94; 0.66–1.36; 0.757
	Allele∶ Trp	32.02%	33.22%	0.515	Allelic	0.95; 0.8–1.12; 0.515
*XRCC1* gene	Arg/Arg	375	340		Additive	1.19; 0.99–1.42; 0.062
Arg399Gln	Arg/Gln	253	241	0.002	Dominant	1.07; 0.86–1.33; 0.551
(rs25487)	Gln/Gln	56	21		Recessive	2.47; 1.48–4.13; <0.001
	Allele∶ Gln	26.68%	23.51%	0.064	Allelic	1.18; 0.99–1.42; 0.064
*XRCC3* gene	Thr/Thr	589	549		Additive	1.64; 1.16–2.32; 0.005
Thr241Met	Thr/Met	93	52	0.017	Dominant	1.67; 1.17–2.38; 0.004
(rs861539)	Met/Met	2	1		Recessive	1.76; 0.16–19.47; 0.644
	Allele∶ Met	7.09%	4.49%	0.005	Allelic	1.63; 1.14–2.33; 0.005

*Abbreviations:* OR, odds ratio; 95% CI, 95% confidence interval.

Under the recessive model, 399Gln/Gln genotype carriers had a 147% increased risk of lung cancer relative to those with 399Arg allele (95% CI: 1.48–4.13; P<0.001). Given the relative sparseness of 241Met/Met homozygotes, except for the recessive model, significance was reached under allelic (OR = 1.63; 95% CI: 1.14–2.33; P = 0.005), additive (OR = 1.64; 95% CI: 1.16–2.32; P = 0.005) and dominant (OR = 1.67; 95% CI: 1.17–2.38; P = 0.004) models.

### Allele combination analysis

To enhance statistical power to detect a firm association, we considered the allele combinations of three examined polymorphisms in this study (Table 3). Overall, frequencies of the most common allele combination Arg-Arg-Thr (in order of Arg194Trp, Arg399Gln, and Thr241Met) were similar between patients and controls (simulated P = 0.145), whereas that of the low-penetrance allele combination Arg-Gln-Met differed significantly (simulated P = 0.001) with 87.1% statistical power to detect this difference. There was no statistical significance for common allele combinations in prediction of lung cancer risk.

**Table 3:** Risk prediction of allele combination of examined polymorphisms for lung cancer before and after adjusting for confounding factors.

Allele combination^†^	Patients	Controls	P_Sim_^*^	OR; 95% CI; P	OR; 95% CI; P^**^
Arg-Arg-Thr	38.77%	42.0%	0.145	Reference	Reference
Trp-Arg-Thr	29.94%	30.91%	0.411	1.03; 0.86–1.25; 0.734	0.99; 0.73–1.36; 0.965
Trp-Arg-Met	2.07%	1.92%	0.261	1.12; 0.54–2.32; 0.759	1.26; 0.48–3.28; 0.64
Arg-Gln-Thr	24.2%	22.21%	0.217	1.18; 0.96–1.45; 0.121	0.88; 0.63–1.22; 0.444
Arg-Arg-Met	2.54%	1.66%	0.079	1.73; 0.84–3.58; 0.139	2.01; 0.61–6.58; 0.252
Arg-Gln-Met	2.48%	0.9%	0.001	3.01; 1.16–7.77; 0.023	3.89; 1.45–8.1; <0.001

*Abbreviations:* OR, odds ratio; 95% CI, 95% confidence interval. * Simulated P values. ** Calculation was performed by adjusting for age, gender, smoking and drinking. ^†^ Alleles were in order of Arg194Trp, Arg399Gln and Thr241Met polymorphisms.

### Interaction analysis

An exhaustive MDR analysis on the possible interaction of three examined polymorphisms is summarized in Table 4. Each best model was accompanied with its testing accuracy, cross-validation consistency and significant level determined by permutation testing. The overall best MDR model included *XRCC1* gene Arg399Gln and *XRCC3* gene Thr241Met polymorphisms, which reinforced the significant results of our single-locus analysis. This model had a maximal testing accuracy of 63.18% and a maximal cross-validation consistency of 10 out of 10. This model was significant at the 0.0175 level.

**Table 4:** Summary of the best combination of three examined polymorphisms by MDR analysis.

Best combination of each model	Cross-validation consistency	Testing accuracy	P
Thr241Met	0.5546	8	0.1859
Thr241Met, Arg399Gln	0.6318	10	0.0175*
Thr241Met, Arg399Gln, Arg194Trp	0.6301	10	0.0273

* The overall best MDR model.

## Discussion

In this study, we sought to investigate the association of three well-characterized nonsynonymous polymorphisms in *XRCC1* and *XRCC3* genes with lung cancer in northeastern Chinese. The principal finding was *XRCC1* gene Arg399Gln and *XRCC3* gene Thr241Met per se were significant contributors to lung cancer. Although all of the common allele combinations of examined polymorphisms were comparable in frequency between patients and controls, we observed potential synergistic effect between these two genes, which reinforced the significant results of our single-locus analysis. To the best of our knowledge, this study represents the first to investigate the interactive impact of *XRCC1* and *XRCC3* genes on lung cancer susceptibility.

Although candidate gene approach cannot replace the genome-wide association study in unraveling the genetic underpinnings of complex disease, it is an important alternative strategy, particularly in the context of adequate sample sizes, ethnic homogeneous populations, and solid biological relevance of the genes concerned. It has been proposed that to generate robust data a large sample size involving more than 1000 subjects in each group is required [12]. Despite that only 684 patients and 602 controls were enrolled in this study, given wide divergence in genetic distributions, a priori power calculation suggested that this study had more than 80% power to detect the loci of realistic effect size. Moreover, our study participants were ethnically homogeneous, and were local residents of Harbin city, where the prevalence of lung cancer is relatively high likely due to the indoor air pollution from the unventilated coal-fueled stoves [13]. In addition, genotypes of examined polymorphisms satisfied Hardy-Weinberg equilibrium in both patients and controls, suggesting the results are unlikely to be biased by genotyping errors or population stratification. Furthermore, selection of *XRCC1* and *XRCC3* genes was based on strong biological, genetic and clinical evidence [2]–[4], [14]–[16], and to enhance the likelihood of identifying disease-causing alleles, nonsynonymous polymorphisms were preferred to those likely to have functionally deleterious consequences.

By far, several meta-analyses have summarized the predisposition of *XRCC1* and *XRCC3* genetic polymorphisms to lung cancer [17]–[20]. Overall, no significant associations were disclosed between all examined polymorphisms and lung cancer under all genetic models, with the exception of contrast of 194Arg/Trp with 194Arg/Arg, which yielded a remarkably protective action [19]. In contrast to the present single-locus results, there was a 2.47-fold increased likelihood that carriers of 399Gln/Gln genotype might develop lung cancer relative to non-carriers. Moreover, Thr241Met mutant allele or genotype was significantly overrepresented in patients, suggesting a potential role of *XRCC3* gene in lung carcinogenesis. Furthermore, beyond the potential importance of individual genetic markers, interaction analysis reinforced the results of our single-locus analysis by identifying a potential synergistic effect between *XRCC1* and *XRCC3* genes. Since the pathophysiological mechanism underlying such interaction is as yet unknown, we speculate that these two genes might interact with each other to play a role in lung carcinogenesis. Nevertheless, considering the limited samplings involved, our results should be interpreted with caution. Because of the complexity of the interactions among gene, these results should be further tested in different races. Therefore, genotyping data from *XRCC1* and *XRCC3* genes, incorporating the haplotype and synergism analytical strategies would facilitate the identification of individuals at high risk of developing lung cancer in future clinical screening.

Some limitations should be acknowledged when interpreting our results. First, the cross-sectional design of this study may preclude comments on causality, and a survival bias could not be excluded. Second, we only focused on three polymorphisms in *XRCC1* and *XRCC3* genes and did not cover the whole genomic sequences of the genes, and thus we may under-evaluate the effects of other genetic markers, Third, data on plasma or tissue XRCC1 and XRCC3 levels are unavailable, which renders us incapable of comparing their levels across genotypes. Fourth, the sample size of this study was not large enough (n = 1286) to draw a firm conclusion, such that our findings need to be validated in an independent population of China and other ethnicities. Thus, we cannot jump to a conclusion until further confirmation of our results is made.

Taken together, our study significantly demonstrated an independent and synergistic contribution of *XRCC1* gene Arg399Gln and *XRCC3* gene Thr241Met polymorphisms to lung cancer susceptibility in northeastern Chinese. For practical reasons, we hope that this study will establish background data for further investigations into the mechanisms of *XRCC1* and *XRCC3* genes and the development of lung cancer.
